# Supplementary material for: Retroactive and graded prioritization of memory by reward
Source: Nat Commun. 2018 Nov 20;9:4886. doi: 10.1038/s41467-018-07280-0 (PMC6244210; doi:10.1038/s41467-018-07280-0)
Supplement: Supplementary file 1 — Supplementary Information [file 41467_2018_7280_MOESM1_ESM.pdf]

Supplementary Methods

Task Instructions by Phase:

For the matrix experiment, participants received the following instructions, presented on the computer screen one or two sentences at a time and read out loud by the experimenter:

Phase 1: maze exploration (incidental encoding) task

> Welcome to the Matrix Game! In this game, you will explore a series of mazes. Your goal is to find the gold coin hidden within each maze. You will be paid $1 bonus for every gold coin that you find. The maze is a 5x5 grid of grey squares. A black frame will indicate where you are in the maze. You can navigate to the space above, below, to the left or right by choosing the corresponding arrow key. You may make a move any time all of the maze spaces are grey. You will have two seconds to make a choice. In each maze you will only get to make a limited number of moves, so if you do not respond in time, you will not have the opportunity to explore as many spaces. The number of moves allowed will vary by maze. After each maze, there will be a short break before the following maze. You should rest during this time. The next maze will begin automatically. Any questions?

Phase 2: surprise recognition memory test

> Welcome back to the Matrix Game! Today, you will play a memory game. Do you remember the pictures you saw in the mazes yesterday? Today, you will see a series of pictures. Your task is to decide whether the picture is OLD, meaning a picture you saw in the maze yesterday, or NEW, meaning a picture you have not seen before. To indicate OLD, select <. To indicate NEW, select >. Then, you will be asked to rate how certain you are about your choice. Respond using the 1, 2, 3 & 4 keys, to indicate your certainty from "guess" to "completely certain". Any questions?

Phase 3: surprise spatial location memory test

> Now, you will play another memory game. This time we will test your memory for the object location within the maze. First, you will see an old object randomly placed in a maze. Your task is to move the object back to the square where you originally found it

using the arrow keys. When you get the object in the correct place, press the space bar. If you cannot remember the exact location, just move the object as close as possible to the original location. Then, just like in the first memory game, we will ask you to rate your confidence, using the 1 to 4 keys. Any questions?

Supplementary Note 1

Phase 2 – subsequent recognition memory, corrected hit rates

To determine if participants' memory performance is above chance, we calculated the corrected hit rate. For subsequent recognition memory tasks in which participants make old/new judgments about their memory for items, corrected hit rate (i.e. hits – false alarms) was used to determine if the participants' memory was above chance, as this criterion measures participants' ability to discriminate between old and new objects by taking into account participants' response bias. Hit rate is defined as the rate at which old objects were correctly identified as old objects; false alarm rate was defined as the rate at which lure objects were incorrectly identified as old objects; and corrected hit rate was defined as hit rate minus false alarm rate.

In all conditions, the mean corrected hit rate was significantly above chance, indicating that participants' recognition memory performance was above chance.

Experiment 1: In the 24-hour condition ($n = 23$), the mean hit rate (SE) was 0.51 ± 0.03, the mean false alarm rate (SE) was 0.28 ± 0.04, and the mean corrected hit rate (SE) was 0.23 ± 0.03 (one sample t-test; $t(22)=7.97$, $p=0.000000062$). In the 15-minute condition ($n = 21$), the mean hit rate (SE) was 0.59 ± 0.03, the mean false alarm rate (SE) was 0.26 ± 0.03, and the mean corrected hit rate (SE) was 0.34 ± 0.03 (one sample t-test; $t(20)=11.44$, $p=0.00000000031$).

Experiment 2 ($n = 21$): We found a hit rate of 0.48 ± 0.03 and a false alarm rate of 0.30 ± 0.03, resulting in a corrected hit rate of 0.18 ± 0.02 (one sample t-test; $t(20)=8.86$, $p=0.000000023$).

Experiment 3 ($n = 32$): We found a hit rate of 0.53 ± 0.02 and a false alarm rate of 0.31 ± 0.02, resulting in a corrected hit rate of 0.22 ± 0.02 (one sample t-test; $t(31)=11.14$, $p=0.0000000000023$).

Experiment 4: In the target detection condition ($n = 27$), we found a hit rate of 0.49 ± 0.02 and a false alarm rate of 0.31 ± 0.03, resulting in a corrected hit rate of 0.18 ± 0.02 (one sample t-test; $t(26)=10.09$, $p=0.000000000018$). In the navigation condition ($n = 27$), we found a hit rate of 0.50

± 0.03 and a false alarm rate of 0.29 ± 0.02, resulting in a corrected hit rate of 0.21 ± 0.03 (one sample t-test; $t(26)=7.42$, $p=0.000000071$). In the working memory condition ($n = 23$), we found a hit rate of 0.49 ± 0.03 and a false alarm rate of 0.32 ± 0.03, resulting in a corrected hit rate of 0.17 ± 0.02 (one sample t-test; $t(22)=7.97$, $p=0.000000063$).

Phase 2 – reward by proximity effect, individual differences

In each of the 24-hour condition recognition memory tests, we find that most participants show a negative reward x proximity effect when the participants' data are analysed individually. In Experiment 1, 78.3% of participants have a negative slope. In Experiment 2, 85.7% of participants have a negative slope. In Experiment 3, 81.3% of participants have a negative slope. In Experiment 4 – target condition, 96.3% of participants have a negative slope. In Experiment 4 – navigation condition, 88.9% of participants have a negative slope. In Experiment 4 – working memory condition, 73.9% of participants have a negative slope.

Supplementary Note 2

Additional potential explanations of the reward by proximity effect

In the following sections, we examine potential modulators of memory that may affect the reward by proximity interaction. In the various analyses described below, we do not find that other variables of interest better explain the data than the reward by proximity interaction.

Phase 2 – subsequent recognition memory, effect of reward one step from maze outcome:

When we test if reward modulates memory for the single object preceding a maze outcome, in most of the datasets, we find that the objects that immediately precede a reward outcome are remembered better than the objects that precede a no reward outcome (multi-level logistic regression; Experiment 1 – 24 hour condition: β=0.52, SE=0.20, z=2.55, CI_{95}=[0.12, 0.91], p=0.013; Experiment 3: β=0.36, SE=0.16, z=2.27, CI_{95}=[0.061, 0.67], p=0.015; Experiment 4 – target condition: β=0.50, SE=0.17, z=2.98, CI_{95}=[0.17, 0.84], p=0.0072; navigation condition: β=0.44, SE=0.17, z=2.60, CI_{95}=[0.11, 0.77], p=0.010). We did not detect an effect of reward for memory on the first step in either Experiment 2, (multi-level logistic regression; β=0.12, SE=0.20, z=0.61 CI_{95}=[-0.27, 0.52], p=0.57) or Experiment 4 – working memory condition (multi-level logistic regression; β=0.26, SE=0.19, z=1.39, CI_{95}=[-0.12, 0.62], p=0.16), although the effects are in same direction.

Phase 2 – subsequent recognition memory, transformed proximity:

One possibility is that the reward proximity effect is being driven entirely by modulated memory only for the objects closest to the reward. If this were the case, we would expect that a log transformed proximity measure would fit the data better. However, when we modelled the data including both mean-centred steps to end interacted with reward and logged steps to end interacted with reward, we found that the reward by proximity interaction reported in the manuscript emerges (multi-level logistic regression; Experiments 1-4 combined: reward X proximity: β = -0.15, SE = 0.034, z = -4.53, p < 0.0004), but we did not detect an interaction between the log transformed proximity and reward (multi-level logistic regression; all 24-hour

datasets combined: reward X log proximity: $\beta = 0.027$, $SE = 0.034$, $z = 0.81$, $p = 0.42$). Consistent with the mean data reward by proximity plots (see Supplementary Figure 1e), this suggests that reward retroactively modulates memory for the preceding sequence of events, not just the object immediately preceding the maze outcome.

Phase 2 – effect of spatial proximity and reward on recognition memory:

Since, during maze navigation, each object is encoded in both a sequential and a spatial location, we were interested to see if rewards retroactively modulated memory for spatially proximal objects (here measured as number of maze steps in any direction to the maze end – minimum one step, maximum eight steps). In each dataset in which we tested memory after 24-hours, we found a reward by spatial proximity interaction such that rewards retroactively modulated memory for spatially proximal objects (or a trend in the same direction). However, one caveat to this analysis is that sequential proximity and spatial proximity are correlated. In models that include both the reward x sequential proximity interaction and the reward x spatial proximity interaction, we detected a reward x sequential proximity interaction but did not detect a reward x spatial proximity interaction.

Experiment 1: We found that reward and spatial proximity interact to predict memory (multi-level logistic regression; reward x spatial proximity: $\beta=-0.086$, $SE=0.032$, $z=-2.67$, $CI_{95} = [-0.15, -0.022]$, $p = 0.011$). However, spatial proximity and sequential proximity were correlated (multi-level regression; spatial proximity ~ sequential proximity: $\beta=0.78$, $SE=0.035$, $t=22.44$, $CI_{95} = [0.71, 0.85]$, $p < 0.0004$). When we controlled for the reward by sequential proximity interaction, we did not detect evidence that reward retroactively modulates memory by spatial proximity (multi-level logistic regression; reward x spatial proximity: $\beta=-0.039$, $SE=0.038$, $z=-1.04$, $CI_{95} = [-0.11, 0.034]$, $p = 0.30$), but we still detected the reward proximity effect (multi-level logistic regression; reward x sequential proximity: $\beta=-0.086$, $SE=0.028$, $z=-2.28$, $CI_{95} = [-0.16, -0.011]$, $p = 0.024$).

Experiment 2: We found a trend such that reward and spatial proximity interact to predict memory (multi-level logistic regression; reward x spatial proximity: $\beta=-0.067$, $SE=0.035$, $z=-1.92$, $CI_{95} = [-0.13, -0.00097]$, $p = 0.044$). However, spatial proximity and sequential proximity were correlated (multi-level regression; spatial proximity ~ sequential proximity: $\beta=0.78$, $SE=0.029$,

t=26.87, CI_{95} = [0.72, 0.83], $p < 0.0004$). When we control for the reward by sequential proximity interaction, we did not detect evidence that reward retroactively modulates memory by spatial proximity (multi-level logistic regression; reward x spatial proximity: β=-.0030, SE=0.040, z=-0.076, CI_{95} = [-0.080, 0.077], p = 0.94), but we still detected the reward proximity effect (multi-level logistic regression; reward x sequential proximity: β=-0.12, SE=0.040, z=-3.09, CI_{95} = [-0.20; -0.040], p = 0.0016;).

Experiment 3: We found a trend such that reward and spatial proximity interact to predict memory (multi-level logistic regression; reward x spatial proximity: β=-0.038, SE=0.027, z=-1.41, CI_{95} = [-0.089, 0.014], p = 0.17). However, spatial proximity and sequential proximity were correlated (multi-level regression; spatial proximity ~ sequential proximity: β=0.79, SE=0.023, t=34.24, CI_{95} = [0.75, 0.84], $p < 0.0004$,). When we controlled for the reward by sequential proximity interaction, we did not detect evidence that reward retroactively modulates memory by spatial proximity (multi-level logistic regression; reward x spatial proximity: β = 0.038, SE = 0.032, z = 1.18, CI_{95} = [-0.024, 0.10], p = 0.24), but we still detected the reward proximity effect (multi-level logistic regression; reward x sequential proximity: β=-0.14, SE=0.032, z=-4.43, CI_{95} = [-0.20, -0.079], $p < 0.0004$).

Experiment 4: We found that reward and spatial proximity interact to predict memory (multi-level logistic regression; reward x spatial proximity: target condition: β=-0.075, SE=0.029, z=-2.59, CI_{95} = [-0.19, -0.063], p = 0.011; navigation condition: β=-0.067, SE=0.030, z=-2.26, CI_{95} = [-0.13, -0.02], p = 0.011; working memory condition: β=-0.056, SE=0.032, z=-1.76, CI_{95} = [-0.12, 0.0070], p = 0.082). However, spatial proximity and sequential proximity were correlated (multi-level regression; spatial proximity ~ sequential proximity: target condition: β=0.72, SE=0.035, t=20.65, CI_{95} = [0.65, 0.79], $p<0.0004$; navigation condition: β=0.74, SE=0.036, t=20.74, CI_{95} = [0.67 0.81], $p < 0.0004$; working memory condition: β=0.80, SE=0.034, t=23.30, CI_{95} = [0.73, 0.87], $p < 0.0004$). When we controlled for the reward by sequential proximity interaction, we did not detect evidence that reward retroactively modulates memory by spatial proximity (multi-level logistic regression; reward x spatial proximity: target condition: β=-0.0094, SE=0.033, z=-0.28 CI_{95} = [-0.073, 0.055], p = 0.78; navigation condition: β=0.0079, SE=0.034, z=0.23, CI_{95} = [-0.057, 0.077], p = 0.84; working memory condition: β=0.015, SE=0.037, z=0.40, CI_{95} = [-0.058, 0.085], p = 0.66), but we still detected the reward proximity effect (multi-level logistic regression; reward x

sequential proximity: target condition: β=-0.13, SE=0.033, z=-4.03, CI_{95} = [-0.20, -0.69], $p < 0.0004$; navigation condition: β=-0.15, SE=0.034, z=-4.44, CI_{95} = [-0.22, -0.085], $p < 0.0004$; working memory condition: β=- 0.13, SE=0.038, z=-3.44, CI_{95} = [-0.21, -0.056], $p < 0.0004$).

<u>Phase 2 – reward proximity effects on recognition memory cannot be explained by recency or primacy:</u>

The reward proximity effect on memory cannot be explained by simple primacy or recency effects (i.e. improved memory for the early or late items in a maze).

Experiment 1: We tested for main effects of primacy (operationalized as steps from the start of the maze) and recency (operationalized as steps from the end of the maze, the proximity measure reported above). In the 24-hour condition, we did not find a main effect of either primacy (multi-level logistic regression; 24-hour condition: proximity to start: β=0.015, SE=0.0094, CI_{95}=[-0.029, 0.0584, p=0.004) or recency (multi-level logistic regression; 24-hour condition: proximity to end: β=-0.16, SE=0.010, CI_{95}=[-0.046, 0.025], p=0.11) on memory. In the 15-minute condition, we found a main effect of primacy (multi-level logistic regression; 15-minute condition: proximity to start: β=-0.024, SE=0.010, CI_{95}=[0.0048, -0.044], p=0.012) on memory but not a main effect of recency (multi-level logistic regression; 15-minute condition: proximity to end: β=-0.016, SE=0.010, CI_{95}=[-0.035, 0.0035], p=0.12).

Experiment 2: We did not detect a main effect of either primacy (multi-level logistic regression; proximity to start: β=0.0029, SE=0.012, CI_{95}=[-0.020, 0.026], p=0.80) or recency (multi-level logistic regression; proximity to end: β=-0.0073, SE=0.010, CI_{95}=[-0.027, 0.012], p=0.48) on memory.

Experiment 3: We did not detect a main effect of either primacy (multi-level logistic regression; proximity to start: β=0.0033, SE=0.027, CI_{95}=[-0.048, 0.060], p=0.88) or recency (multi-level logistic regression; proximity to end: β=-0.038, SE=0.027, CI_{95}=[-0.090, 0.014], p=0.16) on memory.

Experiment 4: We did not detect a main effect of either primacy or recency in any condition (multi-level logistic regression; target detection condition: proximity to start: β=-0.0013, SE=0.0085, CI_{95}=[-0.018, 0.016], p=0.83; target detection condition: proximity to end: β=-0.0026, SE=0.0093, CI_{95}=[-0.021, 0.016], p=0.79; navigation condition: proximity to start: β=0.0029, SE=0.012, CI_{95}=[-0.020, 0.026], p=0.80); proximity to end: β=0.0088, SE=0.0087, CI_{95}=[-0.0083, 0.026], p=0.30; working memory condition: proximity to start: β=-0.011, SE=0.0098, CI_{95}=[-0.049, 0.095], p=0.49); proximity to end: β=0.0057, SE=0.0095, CI_{95}=[-0.071, 0.078], p=0.97).

<u>Phase 2 – no effect of maze length on reward proximity effect on recognition memory</u>

One possible explanation for the reward proximity effect is that the length of the mazes is driving the effect; however, we did not detect evidence of this in our data.

Experiment 1: In the 24-hour condition, we did not find that the reward proximity effect interacts with maze length (multi-level logistic regression; reward x proximity x maze length: β=-0.017, SE=0.061, CI_{95}=[-0.11, 0.14], p=0.81), nor did we find that the reward x proximity x rest duration interaction interacts with maze length (multi-level logistic regression; reward x proximity x rest duration x maze length: β=-0.048, SE=0.061, CI_{95}=[-0.31, 0.16], p=0.58).

Experiment 2: We did not find that the reward proximity effect interacts with maze length (multi-level logistic regression; reward x proximity x maze length: β=-0.039, SE=0.034, CI_{95}=[-0.11, 0.03], p=0.26), nor did we find that the reward x proximity x rest duration interacts with maze length (multi-level logistic regression; reward x proximity x rest duration x maze length: β=-0.059, SE=0.063, CI_{95}=[-0.19, 0.07], p=0.37).

Experiment 3: We did not find that the reward proximity effect interacted with maze length (multi-level logistic regression; reward x proximity x maze length: β=-0.010, SE=0.027, p=0.71); however, only in Experiment 3, we found a trend such that the reward x proximity x rest duration interacted with maze length (multi-level logistic regression; reward x proximity x rest duration x maze length: β=-0.098, SE=0.053, p=0.06).

Experiment 4: In the target detection condition, we did not find that the reward proximity effect interacts with maze length (multi-level logistic regression; reward x proximity x maze length: β=-0.0060, SE=0.030, CI_{95}=[-0.065, 0.052], p=0.81), nor did we find that the reward x proximity x rest duration interacts with maze length (multi-level logistic regression; reward x proximity x rest duration x maze length: β=-0.024, SE=0.056, CI_{95}=[-0.14 0.08], p=0.67). In the navigation condition, we did not find that the reward proximity effect interacts with maze length (multi-level logistic regression; reward x proximity x maze length: β=-0.0057, SE=0.030, CI_{95}=[-0.065, 0.052], p=0.81), nor did we find that the reward x proximity x rest duration interacts with maze length (multi-level logistic regression; reward x proximity x rest duration x maze length: β=-0.083, SE=0.059, CI_{95}=[-0.21, 0.030], p=0.15). In the working memory condition, we did not find that the reward proximity effect interacts with maze length (multi-level logistic regression; reward x proximity x maze length: β=-0.045, SE=0.033, CI_{95}=[-0.11, 0.018], p=0.17), nor did we find that the reward x proximity x rest duration interacts with maze length (multi-level logistic regression; reward x proximity x rest duration x maze length: β=-0.10, SE=0.074, CI_{95}=[-0.06, 0.24], p=0.22).

<u>Phase 2 – the reward proximity effect is not better explained by time elapsed between encoding and maze outcome</u>

Although the jitter was pseudo-randomized across the reward and no reward mazes, one possibility is that the reward proximity effect was confounded by the time elapsed between an object presentation and the maze outcome. When we tested for an interaction of reward and time in seconds from the onset of the end outcome ("time to end") on recognition memory (i.e. replacing proximity with the time elapsed until the outcome), we found results that paralleled the reward proximity reported in the main manuscript (multi-level logistic regression; reward x time to end: Experiment 1 – 24-hour condition: β = -0.11, SE = 0.038, z = -3.12, CI_{95} = [-0.19, -0.043], p = 0.0018; Experiment 2: β = -0.14, SE = 0.033, z = -4.21, CI_{95} = [-.21, -0.075], p < 0.0004; Experiment 3: β = -0.20, SE = 0.061, z = -3.31, CI_{95} = [-0.32, -0.081], p < 0.0004; Experiment 4 – target detection: β = -0.16, SE = 0.029, z = -5.79, CI_{95}=[-0.23, -0.11], p < 0.0004; navigation: β = -0.17, SE = 0.029, z = -5.73, CI_{95}=[-0.23, -.011], p < 0.0004; working memory: β = -0.15, SE = 0.032, z = -4.62, CI_{95}=[-0.21, -0.083], p < 0.0004).

Phase 2 – the reward proximity effect is not confounded by whether the object was presented on the inside or the outside of the maze

Another possibility is that differences in navigational behaviour between the reward and no reward mazes may confound the reward proximity effect. However, participants do not know if a given maze will end in reward or no (low) reward during navigation, and thus, there cannot be systematic difference in navigational behaviour between the two reward conditions.

Another possibility is that different locations within the maze may be more salient. For example, the inside of the maze may have been more salient, and this salience may have biased memory and confounded the reward proximity effect. Here we operationalized inside as the nine squares on the inside of the maze and outside as the 16 squares on the perimeter. We did not find that objects' inside/outside location modulated memory (multi-level logistic regression; inside/outside, coded inside = 1, outside = -1: Experiment 1 – 24-hour condition: $\beta = 0.016$, SE = 0.033, $z = 0.47$, $CI_{95} = [-0.052, 0.081]$, $p = 0.64$; Experiment 2: $\beta = 0.043$, SE = 0.037, $z = 1.16$, $CI_{95}=[-0.029, 0.12]$, $p = 0.25$; Experiment 3: $\beta = -0.023$, SE = 0.032, $z = -0.71$, $CI_{95} = [-0.085, 0.040]$, $p = 0.48$ Experiment 4 – target condition: $\beta = 0.043$, SE = 0.030, $z = 1.43$, $CI_{95} = [-0.015, 0.10]$, $p = 0.17$; navigation condition: $\beta = -0.0060$, SE = 0.030, $z = -0.20$, $CI_{95} = [-0.064, 0.055]$, $p = 0.84$), although in the working memory condition of Experiment 4, we found a trend such that participants were more likely to remember objects from the inside of the maze (multi-level logistic regression; working memory condition: $\beta = 0.060$, SE = 0.033, $z = 1.84$, $CI_{95}=[-0.00084, 0.13]$, $p = 0.050$).

Next, we tested if the reward proximity effect remained even when we accounted for the inside/outside location of encoding. We found that the reward proximity effect persisted in every experiment (multi-level logistic regression; reward x proximity: Experiment 1 – 24-hour condition: $\beta = -0.11$, SE = 0.035, $z = -3.05$ $CI_{95} = [-0.17, -0.037]$, $p = 0.004$; Experiment 2: $\beta = -0.13$, SE = 0.033, $z = -3.78$ $CI_{95} = [-0.19, -0.061]$, $p < 0.0004$; Experiment 3: $\beta = -0.12$, SE = 0.027, $z = -4.46$, $CI_{95} = [-0.17, -0.070]$, $p < 0.0004$; Experiment 4 – target condition: $\beta = -0.14$, SE = 0.029, $z = -4.79$ $CI_{95} = [-0.20, -0.084$, $p < 0.0004$; navigation condition: $\beta = -0.15$, SE = 0.029, $z = -5.03$, $CI_{95} = [-0.20, -0.088]$, $p < 0.0004$; working memory condition: $\beta = -0.12$, SE = 0.032, $z = -3.95$, $CI_{95} = [-0.19, -0.061]$, $p < 0.0004$). In these models, we did not detect a main effect of inside/outside in any

experiment (multi-level logistic regression; inside/outside: Experiment 1 – 24-hour condition: $\beta = 0.011$, $SE = 0.034$, $z = 0.33$, $CI_{95} = [-0.058, 0.075]$, $p = 0.74$; Experiment 2: $\beta = 0.046$, $SE = 0.038$, $z = 1.21$, $CI_{95} = [-0.028, 0.12]$, $p = 0.23$; Experiment 3: $\beta = -0.022$, $SE = 0.032$, $z = -0.70$, $CI_{95} = [-0.085, 0.041]$, $p = 0.48$; Experiment 4 – target condition: $\beta = 0.043$, $SE = 0.029$, $z = 1.47$, $CI_{95} = [-0.014, 0.10]$, $p = 0.14$; navigation condition: $\beta = -0.0034$, $SE = 0.030$, $z = -0.11$, $CI_{95} = [-0.061, 0.056]$, $p = 0.92$), although again we found a trend such that participants were more likely to remember objects from the inside of the maze (multi-level logistic regression; Experiment 4 – working memory condition: $\beta = 0.058$, $SE = 0.033$, $z = 1.77$, $CI_{95} = [-0.0056, 0.12]$, $p = 0.073$). Together, these results suggest that the reward proximity effect is not confounded by the inside/outside encoding location of the object.

Phase 2 – effect of object history by location on recognition memory

We analysed whether the total number of objects encoded in a given location (object history by location) modulated memory in that location. In Experiments 2, 3 and 4, we found that the probability of remembering any given object decreased as the number of total objects encoded in that location increased (multi-level logistic regression; object history by location – minimum = 1 object, maximum = 22 objects, mean = 5.53 objects: Experiment 2: $\beta = -0.035$, $SE = 0.0095$, $z = -3.69$, $CI_{95} = [-0.055, -0.16]$, $p < 0.0004$; Experiment 3: $\beta = -0.037$, $SE = 0.0056$, $z = -6.63$, $CI_{95} = [-0.048, -0.026]$, $p < 0.004$; Experiment 4 – target condition: $\beta = -0.037$, $SE = 0.0093$, $z = -3.99$, $CI_{95} = [-0.056, -0.019]$, $p < 0.0004$; navigation condition: $\beta = -0.025$, $SE = 0.0086$, $z = -2.95$, $CI_{95} = [-0.042, -0.0087]$, $p = 0.0032$; working memory condition: $\beta = -0.052$, $SE = 0.011$, $z = -4.63$, $CI_{95} = [-0.074, -0.031]$, $p < 0.0005$). Additionally, in Experiment 1 – 24-hour condition, we found a trend in the same direction (multi-level logistic regression; object history by location: Experiment 1: $\beta = -0.018$, $SE = 0.010$, $z = -1.76$, $CI_{95} = [-0.037, 0.0017]$, $p = 0.066$).

To answer this question, we tested whether previous objects in location affected memory, and whether this effect was different than an overall effect of time in the full task. In Experiments 2, 3 and 4, we found that as the number of objects previously experienced in any maze location increases, the memory for that object decreased (multi-level logistic regression; object history by location: Experiment 2: $\beta = -0.035$, $SE = 0.0095$, $z = -3.69$, $CI_{95} = [-0.055, -0.16]$, $p < 0.0004$; Experiment 3: $\beta = -0.037$, $SE = 0.0056$, $z = -6.63$, $CI_{95} = [-0.048, -0.026]$, $p < 0.004$; Experiment 4 –

target condition: $\beta = -0.037$, SE = 0.0093, $z = -3.99$, $CI_{95} = [-0.056, -0.019]$, $p < 0.0004$; navigation condition: $\beta = -0.025$, SE = 0.0086, $z = -2.95$, $CI_{95} = [-0.042, -0.0087]$, $p = 0.0032$; working memory condition: $\beta = -0.052$, SE = 0.011, $z = -4.63$, $CI_{95} = [-0.074, -0.031]$, $p < 0.0005$). Additionally, in Experiment 1 – 24-hour condition, we found a trend in the same direction (multi-level logistic regression; object history by location: Experiment 1: $\beta = -0.018$, SE = 0.010, $z = -1.76$, $CI_{95} = [-0.037, 0.0017]$, $p = 0.066$).

However, object history by location was confounded with number of trials encoded, and we found strong correlations between object history by location and encoding trial in each dataset (multi-level regression; Experiment 1 – 24-hour condition: $\beta = 0.049$, SE = 0.0011, $t = 43.56$, $CI_{95} = [0.046, 0.051]$, $p < 0.0004$; Experiment 2: $\beta = 0.049$, SE = 0.0011, $t = 45.97$, $CI_{95} = [0.047, 0.051]$, $p < 0.0004$; Experiment 3: $\beta = 0.049$, SE = 0.00090, $t = 54.29$, $CI_{95} = [0.047, 0.051]$, $p < 0.0004$; Experiment 4 – target: $\beta = 0.047$, SE = 0.00090, $t = 52.07$, $CI_{95} = [0.045, 0.049]$, $p < 0.0004$; navigation: $\beta = 2.81$, SE = 0.076, $t = 37.22$, $CI_{95}=[0.048, 0.053]$, $p < 0.0004$; working memory: $\beta = 0.050$, SE = 0.0013, $t = 37.94$, $CI_{95}=[0.047, 0.052]$, $p < 0.0004$). Therefore, we suspected that the main effect of objects history by location was actually an effect of trials encoded. To test this, we ran models in which both object history by location and encoding trial predicted memory and found that in each model, encoding trial was a significant predictor of memory, such that as encoding trial increased, the likelihood of remembering an object decreased (multi-level logistic regression; encoding trial: Experiment 1 – 24-hour condition: $\beta = -0.0026$, SE = 0.0011, $z = -2.46$, $CI_{95} = [-0.0046, -0.00060]$, $p = 0.0088$; Experiment 2: $\beta = -0.0030$, SE = 0.00098, $z = -3.14$, $CI_{95} = [-0.0050, -0.0012]$, $p = 0.0008$; Experiment 3: $\beta = -0.0030$, SE = 0.00091, $z = -3.33$, $CI_{95} = [-0.0048, -0.0013]$, $p = 0.0008$; Experiment 4 – target: $\beta = -0.0037$, SE = 0.00086, $z = -4.27$, $CI_{95} = [-0.0053, -0.0020]$, $p < 0.0004$; working memory: $\beta = -0.0031$, SE = 0.0010, $z = -3.00$, $CI_{95} = [-0.0051, -0.00098]$, $p = 0.004$). We did not detect a significant effect of encoding trial in Experiment 4 – navigation condition (multi-level logistic regression; encoding trial: $\beta = -0.00079$, SE = 0.00090, $z = -0.88$, $CI_{95}=[-0.0025, 0.00099]$, $p = 0.38$). When we considered the effect of object history, we did not detect any significant effects in these models (multi-level logistic regression; object history: Experiment 1 – 24-hour condition: $\beta = 0.015$, SE = 0.016, $z = 0.98$, $CI_{95} = [-0.016, 0.046]$, $p = 0.34$; Experiment 2: $\beta = 0.0039$, SE = 0.016, $z = 0.25$, $CI_{95} = [-0.027, 0.036]$, $p = 0.85$; Experiment 3: $\beta = -0.0044$, SE = 0.014, $z = -3.039$, $CI_{95} = [-0.034, 0.024]$, $p = 0.75$; Experiment 4 – target: $\beta = 0.012$, SE = 0.014, $z = 0.83$ $CI_{95} = [-0.017, 0.039]$, $p = 0.42$; navigation: $\beta = -0.016$, SE = 0.013, $z = -1.23$, $CI_{95} =$

[-0.042, 0.010], $p = 0.21$; working memory: $\beta = -0.012$ SE = 0.015, $z = -0.77$, $CI_{95} = [-0.042, 0.017]$, $p = 0.44$).

Phase 2 – effect of reward history by location on recognition memory

Additionally, it is possible that the previous reward history of a given location may modulate memory. In Experiment 1 and the target condition of Experiment 4, we did not find that reward history by location, defined as the number of rewards previously experienced in a given object's location, modulated object memory (reward history by location – minimum = 0 rewards, maximum = 4 rewards, mean = 0.26 rewards). In Experiment 1 and two conditions of Experiment 4, we did not find that reward history by location (i.e. for each object the number of rewards previously experienced in that location) modulates object memory (multi-level logistic regression; reward history by location: Experiment 1: $\beta = 0.033$, SE = 0.061, $z = 0.55$, CI_{95} = [-0.091, 0.15, $p = 0.61$; Experiment 4 – target: $\beta = -0.037$, SE = 0.064, $z = -0.59$, $CI_{95} = [-0.16, 0.087]$, $p = 0.58$; working memory: $\beta = 0.022$, SE = 0.057, $z = 0.39$, $CI_{95}=[-0.086, 0.14]$, $p = 0.68$). In Experiments 2, 3 and one condition of Experiment 4, we found a trend such that participants were less likely to remember an object, if more rewards had been previously experienced in that location (multi-level logistic regression; Experiment 2: $\beta = -0.12$, SE = 0.064, $z = -1.94$, $CI_{95} = [-0.25, 0.0090]$, $p = 0.062$; Experiment 3: $\beta = -0.14$, SE = 0.072, $z = -1.92$, CI_{95} = -0.28, 0.0046], $p = 0.058$; navigation: $\beta = -0.098$, SE 0.060, $z = -1.63$, $CI_{95} = [-0.21, 0.021]$, $p = 0.11$). In the working memory condition of Experiment 4, we found the opposite pattern: participants were more likely to remember an object if more rewards had been previously experienced in that location (multi-level logistic regression; working memory condition: $\beta=0.022$, SE=0.057, $z=0.39$, $p=0.070$). This inconsistent pattern of results suggests that previous reward history by location does not reliably modulate subsequent memory.

Next, we tested if the reward proximity effect remained when we accounted for the previous reward history by location and found that the reward proximity effect persisted in every experiment (multi-level logistic regression; reward x proximity: Experiment 1 – 24-hour: $\beta = -0.11$, SE = 0.035, $z = -3.09$, $CI_{95} = [-0.18, -0.043]$, $p = 0.0024$; Experiment 2: $\beta = -0.12$, SE = 0.037, $z = -3.32$, $CI_{95} = [-0.20, -0.049]$, $p < 0.0004$; Experiment 3: $\beta = -0.12$, SE = 0.028, $z = -4.22$, $CI_{95} = [-0.17, -0.062]$, $p < 0.0004$; Experiment 4 – target: $\beta = -0.14$, SE = 0.029, $z = -4.79$, $CI_{95} = [-0.20, -0.084]$, p

< 0.0004; navigation condition: $\beta = -0.15$, $SE = 0.029$, $z = -4.98$ $CI_{95} = [-0.21, -0.092]$, $p < 0.0004$; working memory condition: $\beta = -0.13$, $SE = 0.035$, $z = -3.66$, $CI_{95} = [-0.19, -0.063]$, $p = 0.0008$). In these models, we did not detect a main effect of reward history by location (multi-level logistic regression; reward history by location: Experiment 1 – 24-hour: $\beta = 0.039$, $SE = 0.061$, $z = 0.63$, $CI_{95} = [-0.088, 0.16]$, $p = 0.51$; Experiment 2: $\beta = -0.11$, $SE = 0.064$, $z = -1.79$, $CI_{95} = [-0.25, 0.012]$, $p = 0.066$; Experiment 4 – target: $\beta = -0.031$, $SE = 0.065$, $z = -0.49$, $CI_{95} = [-0.16, -0.098]$, $p = 0.61$; navigation: $\beta = -0.084$, $SE = 0.060$, $z = -1.39$, $CI_{95} = [-0.21, 0.033]$, $p = 0.16$; working memory: $\beta = 0.028$, $SE = 0.057$, $z = 0.49$, $CI_{95} = [-0.085, 0.14]$, $p = 0.64$), except in Experiment 3, in which we detected a negative trend, such that as reward history by location increased, memory decreased (multi-level logistic regression; reward history by location: $\beta = -0.13$, $SE = 0.073$, $z = -1.76$, $CI_{95} = [-0.27, 0.007]$, $p = 0.078$).

Together these analyses suggest that previous reward history does not confound the reward proximity effect.

Supplementary Note 3

Phase 3 – spatial memory measured in steps also retroactively modulated by reward and proximity

The reward proximity effect on spatial memory was not simply a by-product of the chance-corrected memory scoring: we found the same pattern of results when we conduct the same analysis using the uncorrected error score (i.e. the number of steps error between the encoded location and the remembered location; lower error indicates better memory; multi-level regression; Experiments 1-4 combined; 24-hour conditions: reward x proximity: $\beta=0.037$, $SE=0.014$, $t=2.57$, CI_{95} = [0.0094, 0.066], $p = 0.0056$; reward: $\beta=-0.040$, $SE=0.014$, $t=-2.80$, CI_{95} = [-0.068, -0.013], $p = 0.0032$).

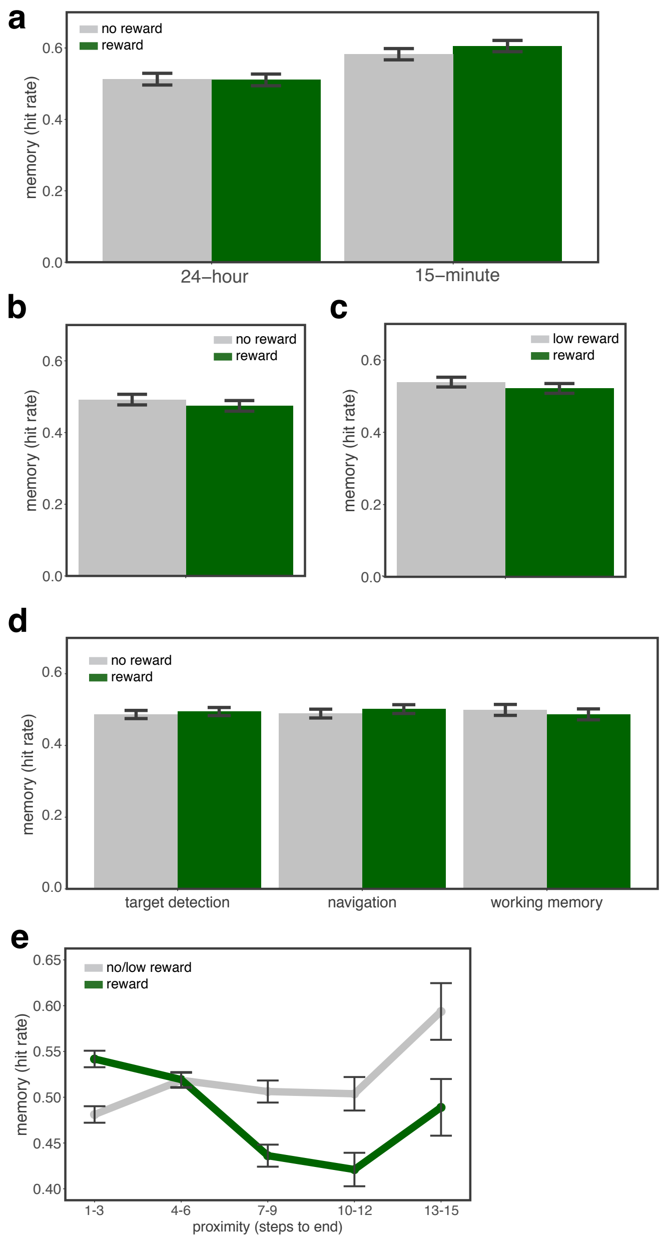

Supplementary Figure 1. *Hit rates by condition*. **(a)** In Experiment 1 (24-hour condition: n = 23; 15-minute condition: n = 21), we found a trend towards a reward by delay condition interaction (multi-level logistic regression; β=-0.083, SE=0.059, CI_{95}=[-0.092, 0.007], p=0.081), such that we detected a significant main effect of reward in the 15-minute condition (multi-level logistic regression; β=-0.082, SE=0.037, CI_{95}=[0.011, 0.15], p=0.023), but not the 24-hour condition (multi-level logistic regression; β=-

0.0037, SE=0.034, CI_{95}=[-0.071, 0.062], p=0.92). **(b)** In Experiment 2 (n = 21), we did not detect a main effect of reward (multi-level logistic regression; β=-0.037, SE=0.033, CI_{95}=[-0.10, 0.029], p=0.25). **(c)** In Experiment 3 (n = 32), we did not detect a main effect of reward (β=-0.038, SE=0.028, CI_{95}=[-0.089, 0.019], p=0.18). **(d)** In Experiment 4 (target detection: n = 27; navigation: n = 27; working memory: n = 23), we did not detect an interaction between distractor condition and reward (multi-level logistic regression; target detection condition vs. navigation condition: β=-0.014, SE=0.041, CI_{95}=[-0.064, 0.096], p=0.73; target detection vs. working memory condition: β=-0.037, SE=0.042, CI_{95}=[-0.12, 0.045], p=0.38). Nor did we detect a main effect of reward in any of the conditions (multi-level logistic regression; target detection condition: β=0.013, SE=0.029, CI_{95}=[-0.042, 0.070], p=0.63; navigation condition: β=0.027, SE=0.029, CI_{95}=[-0.029, 0.086], p=0.35; target detection condition vs. navigation condition: β=-0.024, SE=0.032, CI_{95}=[-0.088, 0.038], p=0.44). **(e)** Mean hit rates by proximity, reward vs. no/low reward. Examining the raw data, we still find a "crossover" pattern, showing an interaction between reward and proximity (multi-level logistic regression; all 24-hour data sets combined: main effect of reward for objects 1, 2, or 3 steps from maze outcome: β=0.26, SE=0.041, z=6.29, p<0.0004; 4, 5, or 6 steps: β=0.0036, SE=0.043, z=-0.084, p=0.93; 7, 8, or 9 steps: β=-0.30, SE=0.052, z=-5.78, p<0.0004; 10, 11, or 12 steps: β=-0.36, SE=0.082, z=-4.33, p<0.0004; 13, 14, or 15 steps: β=-0.45, SE=0.14, z=-3.12, p=0.0018). Error bars represent the standard errors of the difference for each proximity bin.

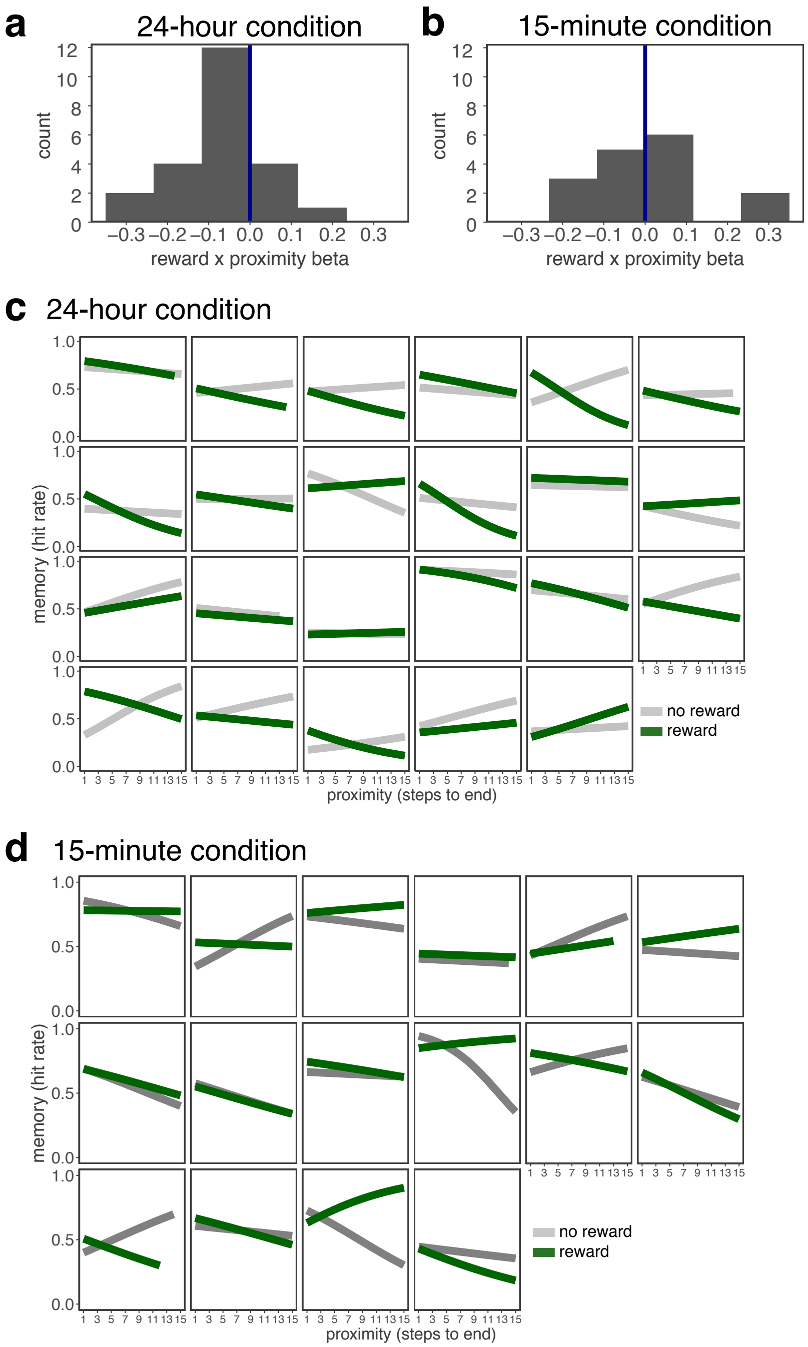

Supplementary Figure 2. *Effect of reward on memory at the individual level.* (Experiment 1). We additionally predicted memory as a function of reward and proximity on participants' data individually. Here, we plot a histogram of the reward x proximity effect for the **(a) 24-hour condition** (n = 23) and **(b) 15-minute condition** (n

= 21). We find that the reward x proximity effect in the 24-hour condition is significantly lower than the reward x proximity effect in the 15-minute condition (regression; β=-0.04, SE=0.016, p=0.014). Post-hoc tests showed that the reward x proximity effect was significantly lower than 0 in the 24-hour condition (one-sample t-test; μ=-0.067, SE=0.022, z=-3.03, p=0.74), but not the 15-minute condition (one-sample t-test; μ=0.01, SE=0.025, z=0.64, p=0.0012). Additionally, we plot the subject-level models for each participant for both the (**c) 24-hour condition** and **(d) 15-minute condition**.

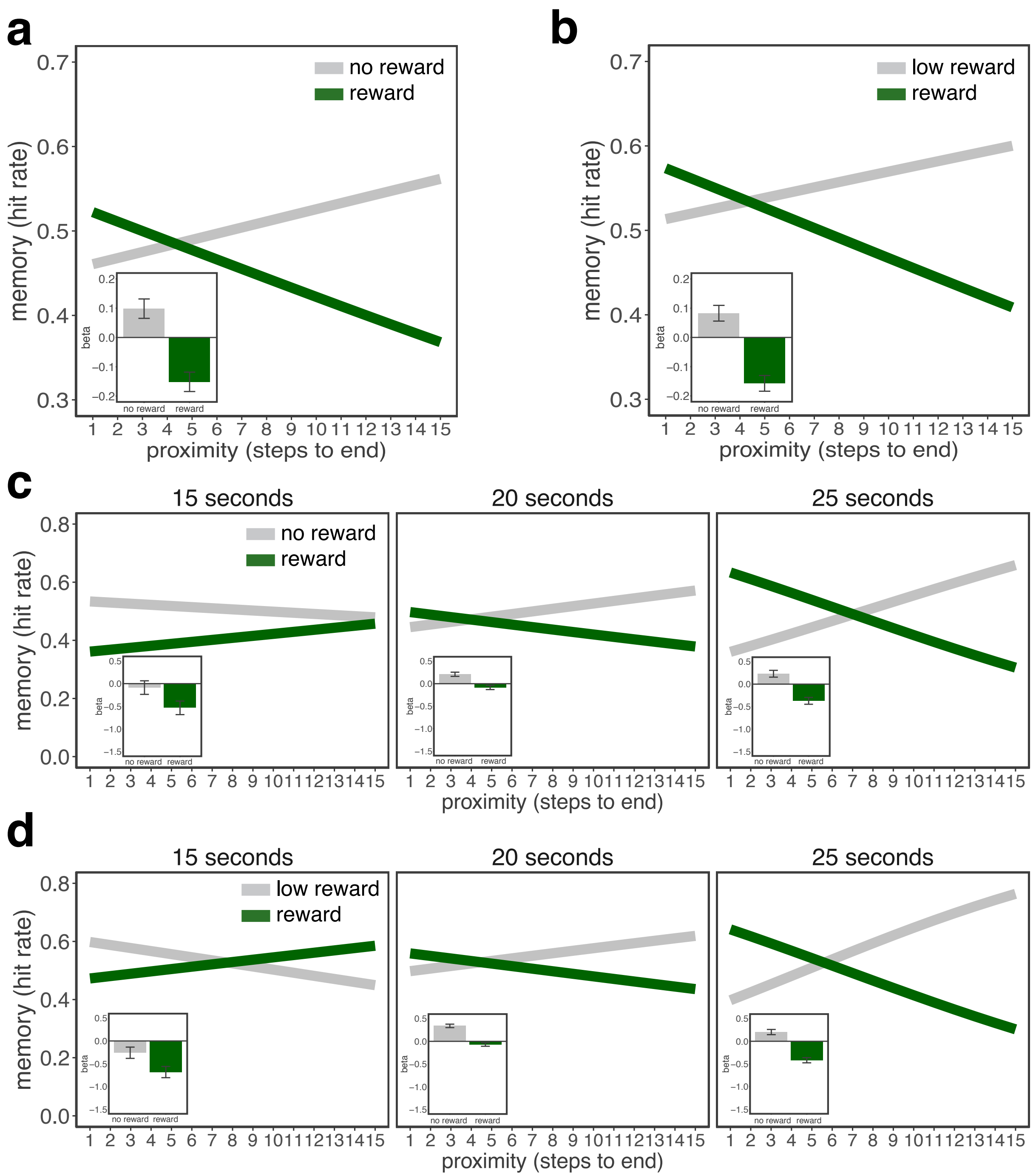

Supplementary Figure 3. *Replication of the reward proximity effect modulated by rest duration.* (Experiments 2 and 3) **(a) Experiment 2.** Reward proximity effect (n = 21). Replicating the 24-hour condition in Experiment 1, rewards retroactively modulated memory, such that participants were more likely to remember objects that were more proximal to the reward. **(b) Experiment 3.** Reward proximity effect (n = 32). In this

version the outcome is always presented within the maze (either $1 or a dime). The results replicate the reward proximity effect. **(c) Experiment 2.** Effect of rest duration. Replicating the 24-hour condition in Experiment 1, we found that the duration of the rest break following each maze modulated the reward proximity effect, such that the interaction was stronger if the rest break following the maze was longer. Again, we find that the model including rest duration explains the data significantly better than the simpler model. **(d) Experiment 3.** Effect of rest duration. Even when the outcome is always presented within the maze (either $1 or a dime), we found that the duration of the rest break following each maze modulated the reward proximity effect, such that the interaction was stronger if the rest break following the maze was longer. The inset depicts the beta coefficients for the reward and no reward conditions; error bars represent the standard error of the reward x proximity interaction.

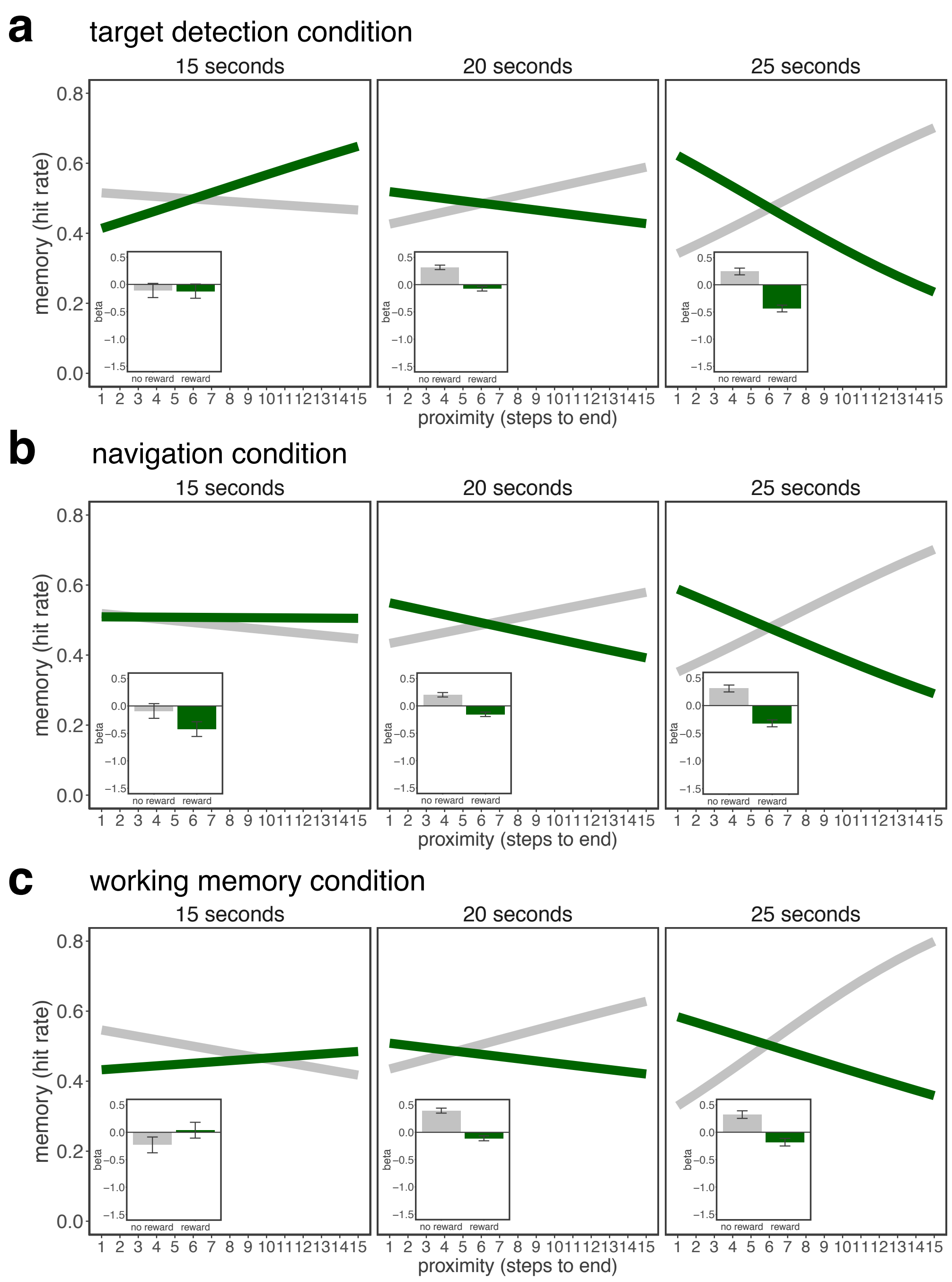

Supplementary Figure 4. *Reward proximity effect increases with longer rest intervals.* (Replication: Experiment 4). **(a)** In the **target detection condition** (n = 27), we found that the duration of the rest break following each maze modulated the reward proximity effect, such that the interaction was stronger if the rest break following the maze was longer (multi-level logistic regression; target detection condition: reward x proximity x

rest duration: $\beta=-0.19$, $SE=0.045$, $CI_{95}=[-0.29, -0.11]$, $p<0.0004$). **(b)** In the **navigation condition** (n = 27), we found that the duration of the rest break following each maze modulated the reward proximity effect, such that the interaction was stronger if the rest break following the maze was longer (multi-level logistic regression; $\beta=-0.13$, $SE=0.039$, $CI_{95}=[-0.21, -0.057]$, $p=0.0016$). **(c)** In the **working memory condition** (n = 23), we found that the duration of the rest break following each maze modulated the reward proximity effect, such that the interaction was stronger if the rest break following the maze was longer (multi-level logistic regression; working memory condition: reward x proximity x rest duration: $\beta=-0.17$, $SE=0.041$, $CI_{95}=[-0.25, -0.090]$, $p<0.0004$). The direct comparison of the three conditions did not reveal a significant difference between the groups (multi-level logistic regression; target detection condition vs. navigation condition x reward x proximity x rest duration: $\beta=-0.061$, $SE=0.055$, $CI_{95}=[-0.048, 0.17]$, $p=0.25$; target detection condition vs. working memory condition x reward x proximity x rest duration: $\beta=0.028$, $SE=0.057$, $CI_{95}=[-0.087, 0.15]$, $p=0.63$) (see **Supplementary Table 3b** for post-hoc tests). The insets depict the beta coefficients for the reward and no reward conditions; error bars represent the standard error of the reward x proximity interaction.

	β	SE	CI_{95}Lower	CI_{95}Upper	p-value
**a. Reward x Proximity x Delay Condition**	-0.061	0.024	-0.11	-0.013	0.012
24-hour Condition: Reward x Proximity	-0.011	0.035	-0.18	-0.036	0.004
Reward: Proximity	-0.16	0.049	-0.25	-0.065	< 0.0004
No Reward: Proximity	0.054	0.045	-0.32	0.14	0.21
15-minute Condition: Reward x Prox	0.014	0.034	-0.051	0.079	0.68
Reward: Proximity	-0.039	0.048	-0.13	0.058	0.41
No Reward: Proximity	-0.067	0.054	-0.17	0.033	0.19

	β	SE	CI_{95}Lower	CI_{95}Upper	p-value
**b. Reward x Proximity x Rest Duration x Delay Condition**	-0.067	0.031	-0.13	-0.0054	0.033
24-hour Condition: Reward x Proximity x Rest Duration	-0.12	0.042	-0.20	-0.036	0.0048
15s Rest Duration: Reward x Proximity	-0.12	0.15	-0.44	0.17	0.44
Reward: Proximity	-0.56	0.30	-1.24	0.027	0.061
No Reward: Proximity	-0.25	0.079	-0.41	-0.099	0.0008
20s Rest Duration: Reward x Proximity	-0.20	0.045	-0.29	-0.11	< 0.0004
Reward: Proximity	-0.11	0.053	-0.21	-0.0062	0.038
No Reward: Proximity	0.29	0.074	0.16	0.46	< 0.0004
25s Rest Duration: Reward x Proximity	-0.22	0.066	-0.36	-0.095	0.0008
Reward: Proximity	-0.26	0.093	-0.46	-0.073	0.008
No Reward: Proximity	0.19	0.094	-0.0077	0.39	0.051
15-minute Condition: Reward x Proximity x Rest Duration	0.18	0.05	-0.081	0.12	0.74
15s Rest Duration: Reward x Proximity	-0.31	0.16	-0.65	-0.0024	0.054
Reward: Proximity	-1.11	0.36	-2.04	-0.46	0.0008
No Reward: Proximity	-0.24	0.092	-0.43	-0.069	0.0048
20s Rest Duration: Reward x Proximity	-0.063	0.055	-0.17	0.045	0.24
Reward: Proximity	-0.044	0.056	-0.16	0.064	0.432
No Reward: Proximity	0.087	0.087	-0.084	0.26	0.31
25s Rest Duration: Reward x Proximity	0.039	0.071	-0.10	0.19	0.58
Reward: Proximity	0.026	0.12	-0.20	0.25	0.80
No Reward: Proximity	-0.059	0.097	-0.25	0.14	0.57

Supplementary Table 1. *Post-hoc analyses for Experiment 1* . Post hoc analyses for the **(a) Reward x Proximity x Delay Condition** interaction and the (**b) Reward x Proximity x Rest Duration x Delay Condition** interaction, including beta values for the interaction (or proximity), standard errors, upper and lower bounds of the 95% confidence intervals, and p-values.

	β	SE	CI_{95}Lower	CI_{95}Upper	p-value
**a. Reward x Proximity**	-0.12	0.033	-0.19	-0.059	0.0008
Reward: Proximity	-0.15	0.048	-0.25	-0.05	0.0008
No Reward: Proximity	0.098	0.052	-0.0053	0.20	0.066

	β	SE	CI_{95}Lower	CI_{95}Upper	p-value
**b. Reward x Proximity x Rest Duration**	-0.14	0.044	-0.23	-0.062	0.0016
15s Rest Duration: Reward x Proximity	-0.23	0.16	-0.56	0.057	0.12
Reward: Proximity	-0.53	0.3	-1.16	0.056	0.072
No Reward: Proximity	-0.087	0.078	-0.24	0.066	0.27
20s Rest Duration: Reward x Proximity	-0.15	0.046	-0.24	-0.054	0.0032
Reward: Proximity	-0.084	0.056	-0.19	0.022	0.12
No Reward: Proximity	0.21	0.074	0.07	0.35	0.004
25s Rest Duration: Reward x Proximity	-0.3	0.076	-0.46	-0.15	< 0.0004
Reward: Proximity	-0.37	0.098	-0.58	-0.18	< 0.0004
No Reward: Proximity	0.23	0.10	0.036	0.44	0.019

	β	SE	CI_{95}Lower	CI_{95}Upper	p-value
**c. Reward x Proximity**	-0.12	0.027	-0.17	-0.071	< 0.0004
Gold Coin: Proximity	-0.16	0.038	-0.24	-0.085	< 0.0004
Dime: Proximity	0.083	0.038	0.0084	0.16	0.029

	β	SE	CI_{95}Lower	CI_{95}Upper	p-value
**d. Reward x Proximity x Rest Duration**	-0.18	0.035	-0.25	-0.11	< 0.0004
15s Rest Duration: Reward x Proximity	-0.19	0.12	-0.45	0.047	0.12
Gold Coin: Proximity	-0.68	0.29	-1.33	-0.12	0.02
Dime: Proximity	-0.26	0.067	-0.4	-0.12	0.0008
20s Rest Duration: Reward x Proximity	-0.2	0.038	-0.28	-13	< 0.0004
Gold Coin: Proximity	-0.071	0.044	-0.16	0.014	0.11
Dime: Proximity	0.34	0.066	0.22	0.48	< 0.0004
25s Rest Duration: Reward x Proximity	-0.31	0.057	-0.43	-0.2	< 0.0004
Gold Coin: Proximity	-0.42	0.082	-0.58	-0.25	< 0.0004
Dime: Proximity					

Supplementary Table 2. *Post-hoc analyses for Experiment 2 and Experiment 3.* Post hoc analyses for **Experiment 2 (a) Reward x Proximity** interaction and the **(b) Reward x Proximity x Rest Duration** interaction. Post hoc analyses for **Experiment 3 (c) Reward x Proximity** interaction and the **(d) Reward x Proximity x Rest Duration** interaction, including beta values for the interaction (or proximity), standard errors, upper and lower bounds of the 95% confidence intervals, and p-values.

		β	SE	CI_{95}Lower	CI_{95}Upper	p-value
**a**	**Reward x Proximity x Distractor Condition (Target Detection vs. Navigation)**	-0.0086	0.041	-0.084	0.074	0.89
	**Reward x Proximity x Distractor Condition (Target Detection vs.Working Memory)**	0.013	0.043	-0.065	0.098	0.68
	Target Detection: Reward x Proximity	-0.14	0.029	-1.95	-0.083	< 0.0004
	Reward: Proximity	-0.15	0.041	-0.23	-0.069	0.0008
	No Reward: Proximity	0.13	0.041	0.050	0.21	< 0.0004
	Navigation Condition: Reward x Proximity	-0.15	0.029	-0.21	-0.089	< 0.0004
	Reward: Proximity	-0.18	0.042	-0.27	-0.098	< 0.0004
	No Reward: Proximity	0.11	0.041	0.35	0.19	0.0064
	Working Memory Condition: Reward x Proximity	-0.13	0.032	-0.19	-0.064	< 0.0004
	Reward: Proximity	-0.11	0.045	-0.19	-0.018	0.022
	No Reward: Proximity	0.15	0.045	0.056	0.24	0.0008

		β	SE	CI_{95}Lower	CI_{95}Upper	p-value
**b**	**Reward x Proximity x Rest Duration x Distractor Condition (Target Detection vs. Navigation)**	0.061	0.055	-0.048	0.18	0.25
	**Reward x Proximity x Rest Duration x Distractor Condition (Navigation vs. Working Memory)**	0.028	0.057	-0.087	0.15	0.63
	Target Detection: Reward x Proximity x Rest Duration	-0.19	0.045	-0.29	-0.11	< 0.0004
	Naviagation: Reward x Proximity x Rest Duration	-0.13	0.039	-0.21	-0.057	0.0016
	Working Memory: Reward x Proximity x Rest Duration	-0.17	0.042	-0.25	-0.09	< 0.0004
	All Distractor Conditions combined:					
	15s Rest Duration: Reward x Proximity	-0.022	0.078	-0.19	0.13	0.75
	Reward: Proximity	-0.18	0.15	-0.49	0.12	0.41
	No Reward: Proximity	-0.14	0.041	-0.22	-0.056	0.0008
	20s Rest Duration: Reward x Proximity	-0.21	0.024	-0.25	-0.16	< 0.0004
	Reward: Proximity	-0.0062	0.17	-0.36	0.31	< 0.0004
	No Reward: Proximity	0.31	0.19	-0.062	0.67	< 0.0004
	25s Rest Duration: Reward x Proximity	-0.30	0.036	-0.37	-0.23	< 0.0004
	Reward: Proximity	-0.32	0.19	-0.42	-0.21	< 0.0004
	No Reward: Proximity	0.29	0.051	0.19	0.15	< 0.0004

Supplementary Table 3. *Post-hoc analyses for Experiment 4.* Post hoc analyses for the **(a) Reward x Proximity x Distractor Condition** interaction and the **(b) Reward x Proximity x Rest Duration x Distractor Condition** interaction, including beta values for the interaction (or proximity), standard errors, upper and lower bounds of the 95% confidence intervals, and p-values. For the post-hoc tests where all of the Distractor Condition groups were combined, group was included as a predictor.
